# Retraction: Appraisal of foliar spray of iron and salicylic acid under artificial magnetism on morpho-physiological attributes of pea (*Pisum sativum* L.) plants

**DOI:** 10.1371/journal.pone.0274233

**Published:** 2022-09-14

**Authors:** The PLOS ONE Editors

The *PLOS ONE* Editors retract this article [1] because it was identified as one of a series of submissions for which we have concerns about authorship, competing interests, and peer review. We regret that the issues were not addressed prior to the article’s publication.

QA agreed with retraction. HN, KS, NZ, MBH, AR, MN, MAQ, MHS, and HMA did not agree with retraction. AAA-H did not reply or could not be reached.
